# From Aryltetralin Anticancer Scaffolds to Host-Directed Bioactivities: A Mechanism-Based Review of Lignans and Neolignans

**DOI:** 10.3390/ijms27167245

**Published:** 2026-08-14

**Authors:** Yuhan Xie, Kun Tang, Paolo Coghi

**Affiliations:** 1 School of Pharmacy, Macau University of Science and Technology, Macau 999078, China; 2 State Key Laboratory of Mechanism and Quality of Chinese Medicine, Macau 999078, China

**Keywords:** podophyllotoxin, medicinal chemistry, biological activities, anticancer activity, structure–activity relationship

## Abstract

Lignans and related neolignans are a diverse class of phenolic natural products with broad biological activities and great potential for pharmaceutical applications. This article summarizes representative subclasses of lignans and compares their antitumor and antioxidant properties from a structural and mechanistic perspective. Aryltetrahydronaphthalene lignans, represented by podophyllotoxin and its semi-synthetic derivatives, provide the clearest baseline for their mechanism of action by disrupting microtubules and inhibiting topoisomerase II. Other subclasses, including dibenzylbutyrolactone, arylnaphthalene, dibenzocyclooctadiene, furofuran lignans, glycosides, and bisphenolic neolignans, exhibit anticancer and redox-regulating effects. Future research should focus on linking structural diversity with validated molecular targets, pharmacokinetic properties, and in vivo efficacy to better define their translational potential.

## 1. Introduction

Lignans and related neolignans are a unique class of phenolic natural products, traditionally defined by the oxidative dimerization of phenylpropane units, and exhibit significant skeletal and stereochemical diversity [1]. In plants, these compounds typically originate from the oxidative coupling of monomeric precursors, where enzymatically generated free radicals undergo regioselective and enantioselective dimerization. Subsequent structural modifications, including hydroxylation, O-methylation, methylenedioxy bridging, glycosylation, and lactoneation, further enrich the structures of these intermediates and generate a variety of lignan and neolignan skeletal families. Therefore, lignans and neolignans exhibit significant structural diversity due to differences in inter-unit linkages, stereochemistry, ring formation, and substitution patterns. Small variations in these features can lead to marked differences in molecular conformation, polarity, metabolism, and biological activity. From a medicinal chemistry perspective, this diversity presents both opportunities and challenges. Differences in stereochemistry and ring systems can endow these compounds with different molecular shapes, oxygen-containing substituents can affect polarity and intermolecular interactions, and these structural features also provide multiple sites for semisynthetic modifications. However, the development potential of lignans and neolignans cannot be simply predicted based on their degree of oxygenation or the presence of phenolic hydroxyl or ether groups. Instead, properties such as water solubility, membrane permeability, and metabolic stability depend on the overall structure of each compound. For example, highly lipophilic compounds such as sesamin and the neolignans magnolol and honokiol have limited water solubility, while absorption of the intact glycoside of glycosylated lignans may be limited, with these glycosides undergoing hydrolysis or microbial transformation in the intestine. Some lignans also undergo extensive metabolism, including demethylation, glucuronidation, and sulfation. Their biological effects are also context-dependent; some compounds exhibit antiproliferative or cytotoxic effects in cancer cells, while others show antioxidant or cytoprotective effects in non-tumor systems. Therefore, the main challenge in the development of lignans and neolignans is not the limitation of a single physicochemical property but rather the need to evaluate and optimize them based on the specific solubility, absorption, metabolic behavior, and biological characteristics of each compound.

Due to their highly flexible aromatic ring substitution patterns and high density of oxygen-containing functional groups, lignans have long been a key research focus in natural product chemistry and a structurally fundamental building block continuously explored in biochemistry and medicinal chemistry. Over the past two decades, extensive review work has cataloged hundreds of new lignans: Teponno et al. summarized 564 lignans and neolignans isolated between 2009 and 2015 [2]; Zálešák et al. further compiled 413 additional examples from 2016 to mid-2018, categorizing them by both structural and bioactive dimensions [3]. Building on this foundation, Wang et al. conducted a comprehensive review of 356 newly reported lignans from 2019 to 2021, systematically discussing their skeletal types, activity profiles, structure–activity relationships, and potential clinical applications [4]. Additionally, Yeung et al. conducted a bibliometric analysis of 10,742 lignan-related publications using the Web of Science database [5]. Results revealed that over 80% of these papers were published after 2000, with nearly 50% appearing post-2010. These data indicate that lignans remain an active area of natural-product research. Most reviews primarily summarize newly isolated compounds, structural types, and bioactivity profiles; however, less attention is paid to how specific structures are associated with biological mechanisms. For example, it remains unclear which structural features are associated with shifts between different biological activities, which modifications might alter the primary mechanism of action, and how the diversity of natural lignans can be leveraged to guide medicinal chemistry design. Although podophyllotoxin and its semi-synthetic derivatives provide useful structure–activity relationships, these findings remain scattered across different disease models and experimental systems rather than being discussed within a unified medicinal chemistry framework. From a biosynthetic perspective, the diversity of lignan structures provides a theoretical basis for cross-skeletal comparisons. Lignans are mainly derived from phenylalanine. Phenylalanine is first converted to cinnamic acid by phenylalanine ammonia-lyase and then further transformed through steps such as hydroxylation, O-methylation, CoA activation, reduction, and dehydrogenation to generate C6-C3 monolignan precursors [6]. Coniferyl alcohol plays an important role in the formation of lignan structures. Oxidases can convert coniferyl alcohol into phenoxy radicals, which are then guided by dirigent proteins to undergo regioselective and stereoselective coupling to form pinoresinol, thus forming the early 8-8′ lignan backbone [7]. Subsequently, pinoresinol and related intermediates can undergo further reduction and enzymatic modification reactions, resulting in various lignan subclasses, including cytochrome P450-mediated oxidation, O-methylation, glycosylation, lactoneation, and the formation of methylenedioxy bridges [8]. In sesame, (+)-pinoresinol is sequentially converted to (+)-piperitol and (+)-sesamin through CYP81Q1-mediated formation of methylenedioxy bridges. (+)-Sesamin can then undergo CYP92B14-mediated oxidative rearrangement to generate (+)-sesamolin and (+)-sesaminol [9]. Sesaminol is subsequently subjected to regioselective sequential glycosylation by UDP-dependent glycosyltransferases to produce sesaminol glycosides, including sesaminol triglucoside [10]. This biotransformation process elucidates the reason why lignans possess a rich stereochemical structure and multiple modifiable sites. It further explains the potential relationship between different conformational preferences, physicochemical properties, and biological activities.

**Figure 1:**
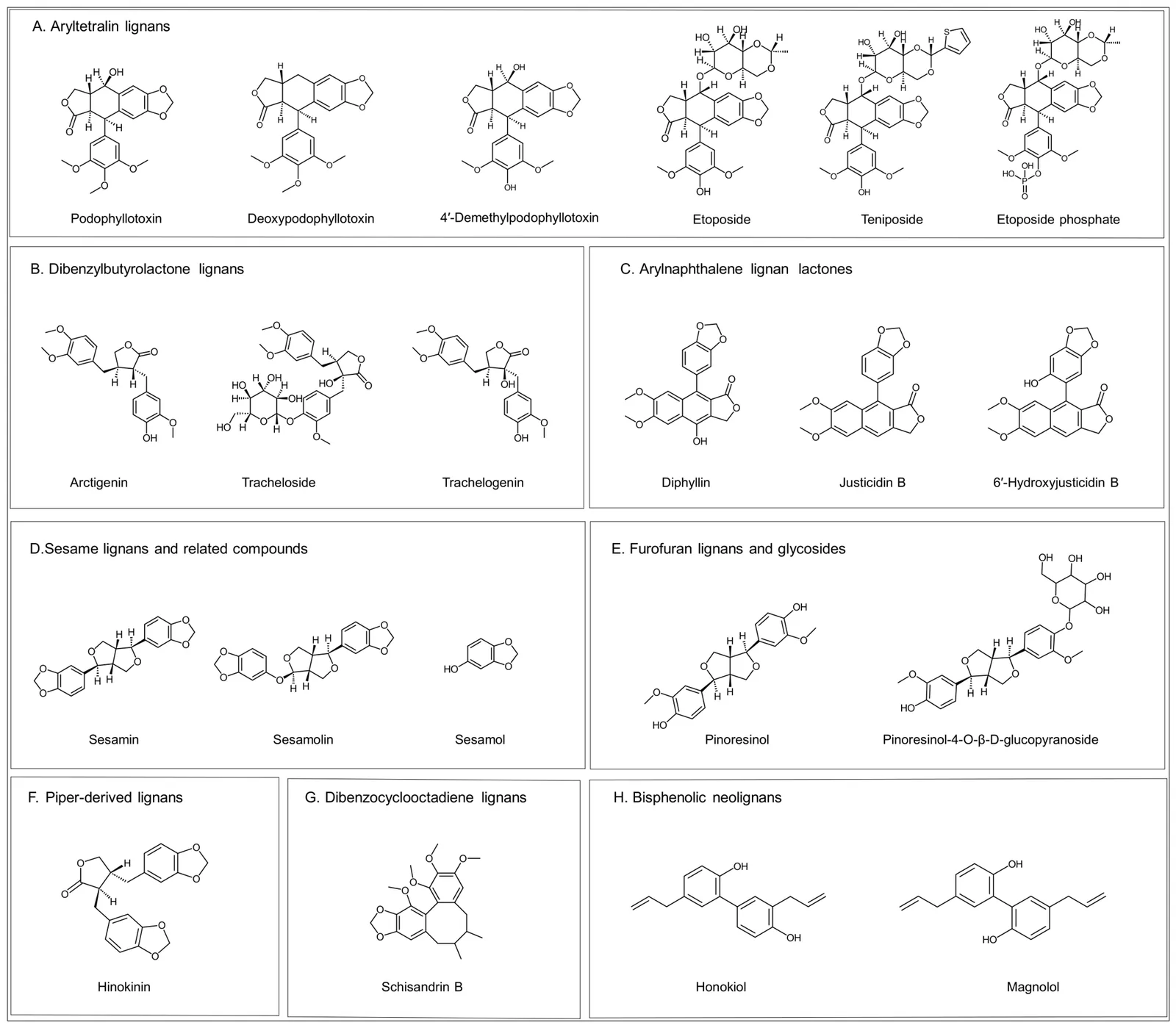
Chemical structures of representative aryltetralin lignans and other lignans and neolignans discussed in this review.

Therefore, combining mechanistic studies with comparisons of different skeletons remains crucial for exploring lignans. Further cross-skeletal comparative studies are still needed. Lignans are considered dimers formed by the oxidative coupling of two C6-C3 phenylpropane units. Based on the topological structure of the dimer core, lignans can be divided into several major subclasses, including dibenzylbutane, dibenzylbutyrolactone, furan, furofuran or tetrahydrofuran, benzofuran, arylnaphthalene, aryltetrahydronaphthalene, dibenzocyclooctadiene, norlignan, and other related structural types [11] (Figure 1).

**Figure 2:**
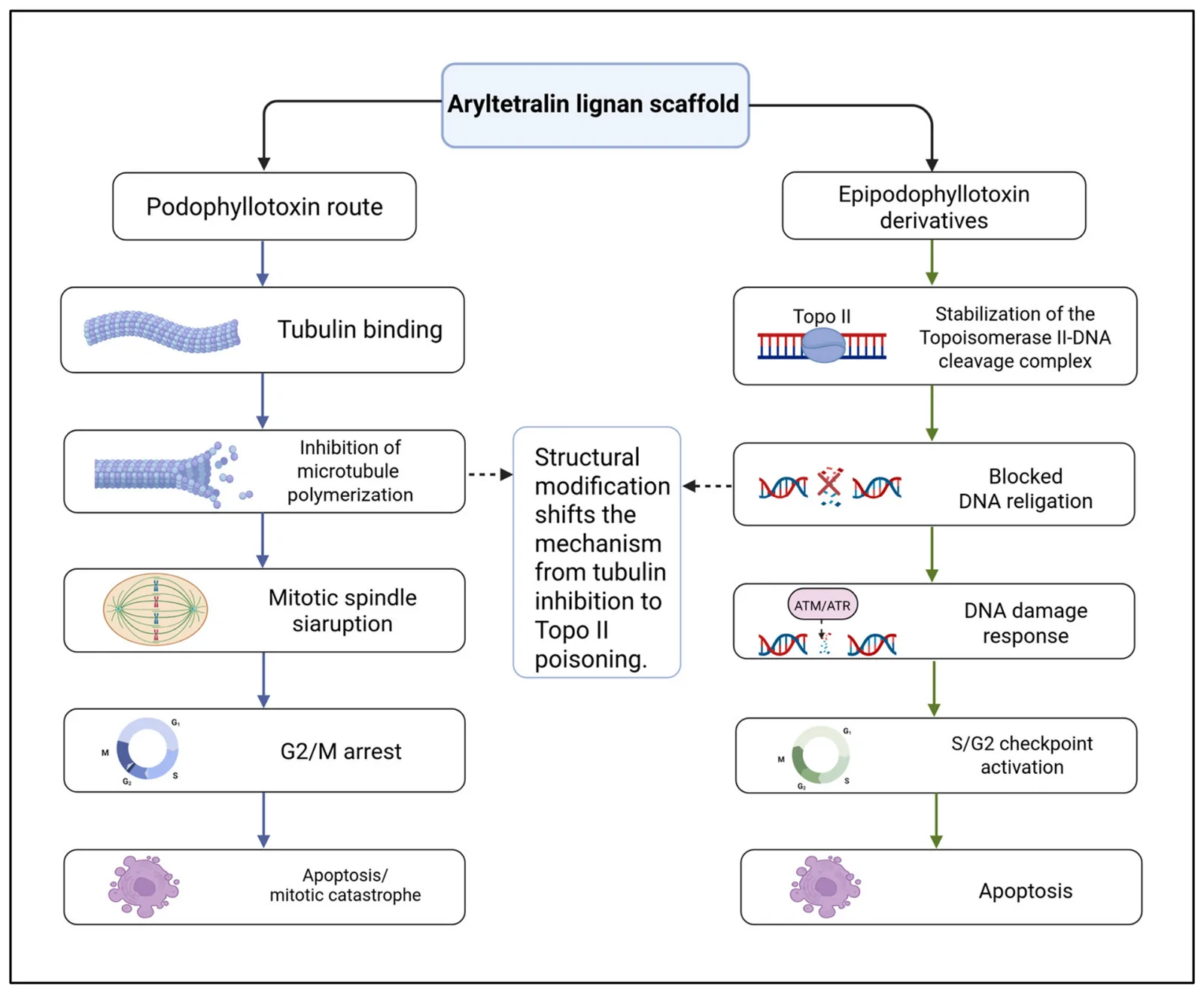
Mechanistic divergence of podophyllotoxin-type aryltetralin lignans. The above figure was created in BioRender: https://BioRender.com/u8xkx2e.

Lignans are dimeric compounds formed by two phenylpropane units linked via β-β′ bonds, whereas neolignans include compounds linked through other mechanisms, such as the common 8-O-4′, 8-5′, and 7-O-3′ bonds. When discussing structural diversity and bioactivity in natural product and pharmacological research, lignans and neolignans are often discussed together. Among these structural subclasses, aryltetralin lignans stand out. They are among the most extensively studied lignans in pharmacology and are closely related to medicinal chemistry. Based on their skeletal characteristics, aryltetralin lignans can be broadly classified into two categories. The first category consists of podophyllotoxin-type aryl-tetrahydronaphthyl lignans, represented by the classic aryltetralin lactone skeleton. This category includes podophyllotoxin, deoxypodophyllotoxin, 4′-demethylpodophyllotoxin, their glycosides, and related semisynthetic derivatives. The second category consists of non-podophyllotoxin-type aryltetralin lignans, which differ from the typical podophyllotoxin skeleton in terms of aromatic substitution patterns, ring number, or bridging configurations. Examples include aryltetralone/aryltetralol derivatives from the genus *Holostylis*, polycyclic aryl-tetrahydronaphthalenes from the genus *Machilus*, and Tsugacetal or aryl-tetrahydronaphthalene structures reported from plants of the genera *Forsythia*, *Phyllanthus*, and *Epimedium*. Podophyllotoxin is a prime example of the connection between natural products and clinical drug development [12,13,14]. Its parent structure comprises multiple stereocenters and a rigid polycyclic core. More importantly, podophyllotoxin-related derivatives provide a clear direction for transforming natural products into clinical anticancer drugs. Classic semisynthetic derivatives, such as etoposide, modify the original backbone by introducing sugar-containing substituents at key positions, thereby altering their interactions with the target and providing valuable experience for further structural optimization. Within the lignan family, different substitution patterns result in diverse biological activities, including antioxidant, anti-inflammatory, antibacterial, and antitumor effects, as well as influences on transport proteins or enzymes [15]. This diversity may be related to the balance between the rigidity and flexibility of the lignan skeleton and the modifiable substitution sites. For example, relatively flexible dibenzylbutane derivatives and more rigid polycyclic structures interact with protein targets in different ways. Furthermore, phenolic hydroxyl, methoxy, methylenedioxy, glycosidic, and other substituent positions provide opportunities for derivatization, and the introduction of functional groups can improve or tune solubility, metabolic stability, selectivity, or targeting. Aryltetralin lignans, represented by podophyllotoxin and its semisynthetic derivatives, provide a particularly clear example of how structural modification can alter the pharmacological behavior of a natural-product scaffold. Podophyllotoxin mainly acts by binding to tubulin and disrupting microtubule assembly, whereas the clinically used epipodophyllotoxin derivatives etoposide and teniposide primarily act as topoisomerase II poisons [16,17]. Thus, closely related aryltetralin scaffolds can produce distinct mechanisms of action through changes in substitution and molecular interactions. This mechanistic divergence is summarized in Figure 2 and makes the podophyllotoxin family a useful reference for understanding how structural modification of lignans can influence target engagement and biological activity. The detailed anticancer mechanisms and related evidence are discussed in Section 2.1. Research on lignans often spans multiple levels, including natural product isolation, initial activity screening, mechanism inference, target validation, and animal or preclinical evaluation. When discussing biological activity in this paper, we will strive to differentiate between varying levels of evidence strength: We will prioritize model results with clear effect sizes and well-defined control settings, further emphasizing studies with direct mechanistic pathways or target-level evidence. Data limited to single-point inhibition, coarse phenotypes, or lacking critical controls will be incorporated primarily as potential clues rather than definitive conclusions. This tiered presentation ensures comparisons between lignan lineages are grounded in comparable evidence bases. However, another equally valid observation emerged during the writing of this review: when the scope is strictly confined to aryltetralin lignans, systematic evidence supporting their efficacy in antibacterial and antioxidant applications tends to be more fragmented and less comprehensive, except for antitumor research closely associated with the podophyllotoxin lineage.

Conversely, other lignan families, including dibenzylbutyrolactone, dibenzocyclooctadiene, arylnaphthalene, and furofuran lignans, as well as selected lignan glycosides and neolignans, provide complementary evidence across different biological activities and mechanisms. Therefore, this review uses aryltetralin lignans as a mechanistic reference scaffold while incorporating representative compounds from other structural branches to provide a broader cross-skeletal comparison of anticancer and antioxidant activities.

## 2. Biological Activities

Research on the biological activities of lignans and related neolignans spans a broad spectrum, though evidence density varies across different skeletal branches and disease areas. To facilitate cross-comparisons and subsequent discussions, this section will systematically review activity types in the following order: antitumor and antioxidant. Within each section, we will first briefly outline commonly used experimental indicators and typical activity manifestations for that direction, followed by discussions centered on several representative compounds and structural lineages. This approach will clearly highlight the functional advantages, limitations, and potential development opportunities across different lignan branches.

### 2.1. Anticancer Activity

**Figure 3:**
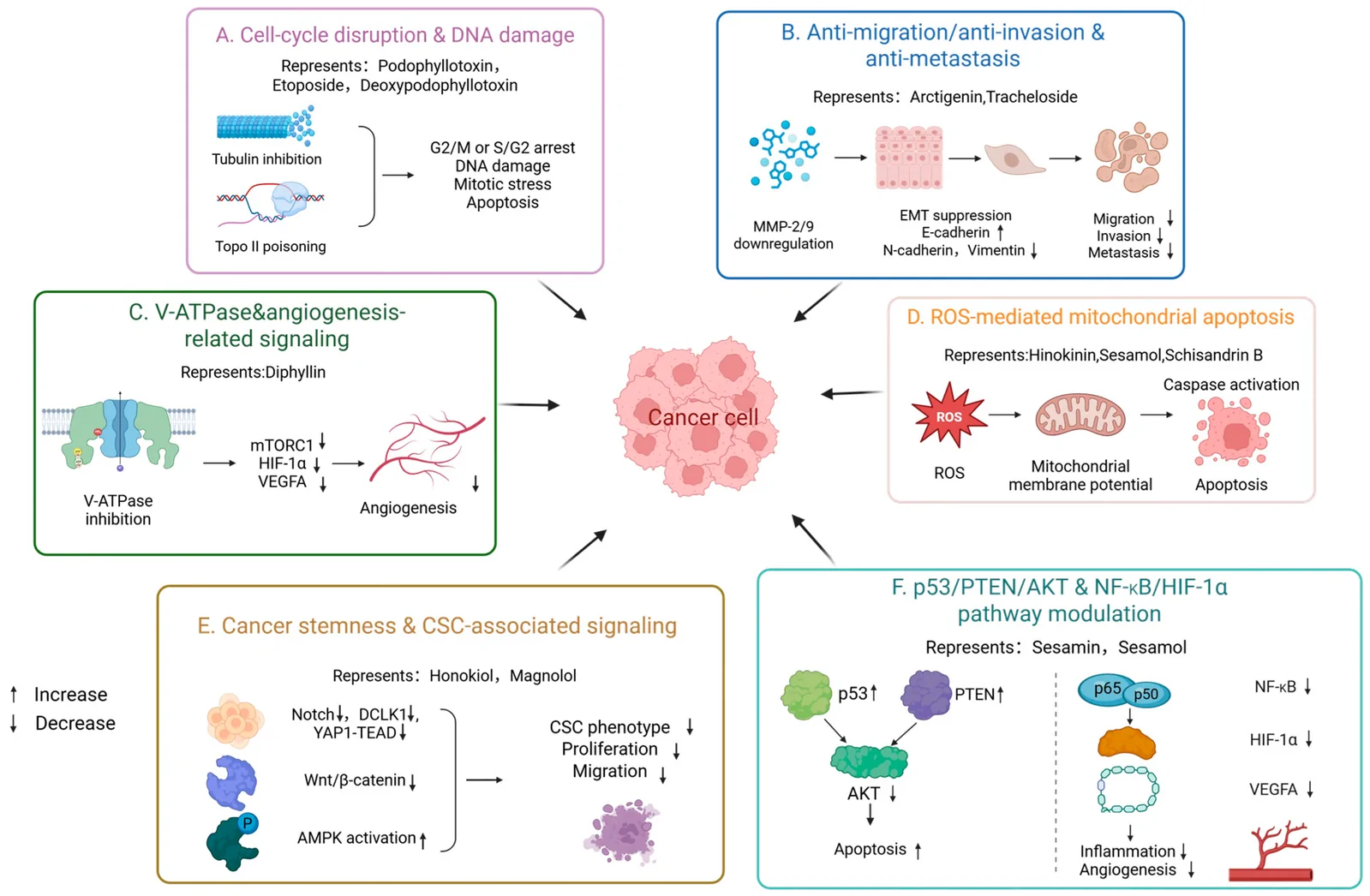
Anticancer mechanisms of representative lignan and neolignan branches. This schematic summarizes the main anticancer mechanisms of representative lignans and related neolignans, including cell-cycle disruption, DNA damage, apoptosis, EMT/MMP regulation, angiogenesis-related signaling, ROS-mediated mitochondrial injury, and suppression of cancer stemness-associated pathways. Created in BioRender. https://BioRender.com/d4a4c9m.

Cancer remains one of the major global public health burdens. In 2022, there were nearly 20 million new cancer cases worldwide, resulting in approximately 9.7 million deaths [18]. Despite ongoing advances in surgery, radiotherapy, and systemic therapies, there is a persistent need to supplement the drug pipeline with agents featuring novel mechanisms of action or improved therapeutic windows. Among the semisynthetic drugs developed from the podophyllotoxin family, etoposide and its water-soluble prodrug etoposide phosphate are indicated for treating small cell lung cancer and refractory/relapsed testicular tumors. Teniposide, also belonging to this family, is indicated for induction therapy of refractory pediatric acute lymphoblastic leukemia in combination with other chemotherapeutic agents. Additionally, the approved indications for etoposide phosphate may vary across different regions. For instance, some regions also list Hodgkin lymphoma, non-Hodgkin lymphoma, and acute leukemia, reflecting the broad application of this class of drugs within multi-agent regimens. Regarding the parent compound podophyllotoxin, its antitumor effects are most commonly attributed to a microtubule-targeting mechanism. Early studies have demonstrated that podophyllotoxin significantly inhibits microtubule assembly in vitro, leading to cell accumulation at the G2/M phase and subsequent outcomes such as apoptosis or mitotic catastrophe. Etoposide and teniposide are generally classified as Topo II poisons, increasing the level and/or lifetime of Topo II-DNA cleavage complexes and preventing DNA religation. These molecules stabilize the Topo II-DNA cleavable complex and inhibit the rejoining of broken DNA, leading to the accumulation of DNA damage signals and triggering cell death [19,20]. Deoxypodophyllotoxin (DPT) provides in vivo evidence closer to that of its natural homologue. Khaled et al. reported the antitumor effect of DPT after forming a complex with 2-hydroxypropyl-β-cyclodextrin (HP-β-CD) in the MDA-MB-231 breast cancer xenograft model. The comparative conclusions presented indicate that the in vivo antitumor effect of DPT-HP-β-CD (20 mg/kg) surpasses that of etoposide (VP-16) and docetaxel at equivalent doses [21]. It suggests that DPT is not merely a synthetic precursor but may also possess considerable in vivo pharmacological potential. The aryltetralin scaffold contains several chemically accessible positions for modification, including substituents around the aromatic rings and the C-4 region. Botta et al. previously summarized key knowledge frameworks regarding the structural types, pharmacological activities, and biotransformation of this lignan class [22]. Recent comprehensive reviews have further integrated these insights with modern synthetic, semi-synthetic, and biosynthetic modules, emphasizing that site editing serves not only to enhance potency but also to rebalance solubility, metabolic stability, and safety margins [23,24]. The major anticancer mechanisms associated with the representative lignan and neolignan branches discussed below are summarized in Figure 3.

Beyond classic site modifications, one recent trend involves deeper skeletal redesign, exemplified by aza-podophyllotoxin derivatives. Tailor et al. reported the activity of a novel aza-podophyllotoxin derivative in a triple-negative breast cancer model, proposing that it induces cell death by activating AMPK and pathways associated with oxidative phosphorylation while demonstrating antitumor potential in in vivo models [25]. Aryltetralin lignans represent one of the lignan families with the most robust evidence base in the antitumor field. However, future gains are more likely to stem from selective targeting, exposure control, and resistance countermeasures rather than solely pursuing further reductions in IC_50_ values. Representative quantitative anticancer evidence for the major lignan and neolignan branches discussed in this review is summarized in Table 1.

#### 2.1.1. Dibenzylbutyrolactone

The podophyllotoxin lineage offers cases with well-defined targets and complete chains of evidence, while other lignan lineages resemble activity profiles with richer phenotypes but more dispersed mechanisms. To complement this benchmark and reflect the broader landscape of lignan bioactivity, representative non-aryltetralin scaffolds are summarized below, focusing on studies that connect in vitro phenotypes to in vivo endpoints. Among these, dibenzylbutyrolactone lignans have attracted attention for their consistent anti-migratory and anti-invasive readouts in colorectal cancer models [48]. Arctigenin (ARC), a dibenzylbutyrolactone lignan, demonstrated anti-metastatic activity in colorectal cancer models. In CT26, MC38, and SW620 cells, ARC reduced cell viability and suppressed migration and invasion, accompanied by changes in MMP-2/MMP-9 and EMT-related markers. In a CT26 experimental lung metastasis model, oral administration of ARC (50 mg/kg/day for 2 weeks) reduced pulmonary tumor nodules by approximately 30%. Tracheloside (TCS) is another DBBL-type lignan glycoside. In vitro experiments demonstrate that TCS reduces CRC cell viability, with more pronounced inhibition observed in mouse-derived CT26 cells [29]. Mechanistically, TCS induces cell cycle arrest at the G0/G1 phase, accompanied by p16 upregulation and cyclin D1/CDK4 downregulation. Concurrently, it triggers a mitochondrial-mediated apoptotic pathway characterized by endogenous apoptosis signals, thereby increasing the proportion of apoptosis and collectively inhibiting tumor cell proliferation. In vivo, oral administration of TCS (25–50 mg/kg) in a mouse lung metastasis model established via CT26 tail vein injection reduced the number of pulmonary nodules without significant body weight changes, consistent with in vitro findings. Pro-apoptotic protein alterations were similarly observed in lung tissues. At low doses with no apparent cytotoxicity, TCS significantly inhibited CRC cell migration and invasion in scratch healing and Transwell assays. Overall, TCS demonstrated anti-CRC activity in both cellular and animal models, exhibiting dual inhibitory effects on proliferation and invasion-related phenotypes. An in vitro study reported that trachelogenin, a dibenzyl butyrolactone-type lignan isolated from *Combretum fruticosum*, exhibits significant antitumor activity in human colon cancer cells HCT-116. At the cytotoxicity level, the authors employed the MTT assay to test 72 h exposure across multiple tumor/non-tumor cell lines. Results demonstrated that trachelogenin exhibited significant inhibitory effects on tumor cells (IC_50_ ranging from 0.8–32.4 μM) while showing no apparent toxicity to various non-tumor cell lines (IC_50_ > 64 μM), suggesting a certain degree of tumor selectivity. Subsequent reviews summarizing this work noted that trachelogenin induces the appearance of acidic vesicular organelles (AVOs) and autophagic vacuoles in HCT-116 cells at concentrations as low as 5–10 μM [30,49]. This effect is accompanied by Beclin-1 upregulation and enhanced conversion of LC3-I to LC3-II, further underscoring its autophagy-mediated antiproliferative mechanism. Thus, dibenzylbutyrolactone lignans represent a phenotype-rich but mechanistically more dispersed antitumor branch, with current evidence pointing particularly toward anti-migratory, anti-metastatic, pro-apoptotic, and autophagy-related effects.

#### 2.1.2. Arylnaphthalene Lignan Lactones (Justicidin/Diphyllin)

Diphyllin is a small molecule reported as a novel V-ATPase inhibitor [32,33]. In vitro studies on esophageal carcinoma cells TE-1 and ECA-109 demonstrated that diphyllin induces significant antitumor phenotypes at submicromolar concentrations: CCK-8 assays (48 h) yielded IC_50_ values of ≈0.2058 μM for TE-1 cells and ≈0.2792 µM for ECA-109 cells, indicating potent proliferation inhibition in both cell lines. Cell cycle analysis further demonstrated that diphyllin induced S-phase arrest in both cell lines, exhibiting distinct cell cycle regulatory properties. Concurrently, it reduced cellular V-ATPase activity and decreased mTORC1/HIF-1α/VEGF-C transcription levels, thereby attenuating tumor cells’ pro-angiogenic signaling output to endothelial cells. Given prior studies suggesting diphyllin’s potential to inhibit V-ATPase but limited evidence in esophageal cancer, the authors integrate the observed reductions in V-ATPase activity, mTORC1/HIF-1α/VEGF-C transcription, and VEGF-C secretion to propose the following hypothesis: diphyllin may inhibit VEGF-C-related output by suppressing V-ATPase, thereby inhibiting mTORC1 signaling and downregulating HIF-1α, ultimately attenuating biological processes associated with metastasis and tumor angiogenesis. However, the key causal chain requires further validation through more in-depth mechanistic and in vivo studies. Diphyllin and arylnaphthalene lignan lactones derived from *Justicia procumbens*, along with the representative monomer justicidin B, share the same arylnaphthalene lignan lactone skeleton [34]. In contrast, arylnaphthalene lignan lactones from *J. procumbens* exhibit differential cytotoxic activity against leukemia K562 cells, including 6′-hydroxy justicidin B, 6′-hydroxy justicidin A, justicidin B, and two methyl ether derivatives CME and TEME. MTT assay (48 h) revealed 6′-hydroxy justicidin B (IC_50_ = 20 μM) as the most potent, followed by 6′-hydroxy justicidin A (43.9 μM) and justicidin B (45.4 μM). Flow cytometry and Annexin V/PI results indicated that 6′-hydroxy justicidin B/6′-hydroxy justicidin A/justicidin B induced an increase in apoptosis-associated the sub-G0 population and elevated the proportion of early and late apoptosis stages. Active caspase-3 detection confirmed caspase-3 activation, supporting their role in triggering caspase-dependent apoptosis mechanisms. The authors further established clear structure–activity relationships: hydroxyl substitution at positions C-1 and C-6′ significantly enhances antiproliferative/apoptogenic effects, whereas methoxy substitution at C-1 diminishes activity [35]. Future optimization should focus on preserving key hydroxyl sites. Justicidin B serves as a representative compound of this class, and literature evidence corroborates the caspase-dependent apoptotic phenotype observed in K562 cells: in other leukemia cell lines (e.g., HL-60), justicidin B was used to evaluate its apoptotic effects, with MTT-measured IC_50_ values of approximately 3.6 μM (24 h), 2.3 μM (48 h), and 0.9 μM (72 h). The primary pathway involved is the mitochondrial intrinsic route, centered on the caspase-9/3 pathway. Comparisons with *J. procumbens* series compounds reveal a common feature: This class of arylnaphthalene lignan lactones typically induces apoptosis as the primary endpoint, primarily through the endogenous mitochondrial signaling pathway rather than the extrinsic pathway.

#### 2.1.3. Sesame-Derived Lignans and Related Phenols

Sesame-derived lignans and related phenols, represented by sesamin, sesamolin, sesamol, and sesaminol, constitute a class of multidimensional anticancer compounds exhibiting anti-proliferative, pro-apoptotic, anti-inflammatory, anti-metastatic, anti-angiogenic, and autophagy-modulating effects [50,51]. In multiple solid tumor cell models, sesamin consistently exhibits proliferation inhibition at the μM level. In cervical cancer models, 75–150 μM sesamin dose-dependently suppressed HeLa/SiHa proliferation while significantly increasing the sub-G1 and apoptotic fractions (approximately 30–40%). Mechanistically manifested as increased p53 (Ser15/Ser46) phosphorylation, upregulation of PUMA/Bax/PTEN, and decreased AKT (Ser473) phosphorylation, thereby promoting cell death through the p53-PTEN-AKT tumor suppressor network [36]. Studies in colorectal cancer also demonstrate that sesamin inhibits hypoxia-induced angiogenesis in vitro models. It achieves this by suppressing the phosphorylation of IκBα, thereby limiting the promotion of HIF-1α transcription by NF-κB p65, which consequently reduces VEGFA expression [37]. Sesamin also commonly acts on MMP and MAPK. In head and neck squamous cell carcinoma cells, non-cytotoxic concentrations ranging from 0 to 40 μM can inhibit migration/invasion and downregulate MMP-2, accompanied by reduced p38 and JNK phosphorylation. Additionally, in PD-L1-high triple-negative breast cancer MDA-MB-231 cells, sesamin reduces PD-L1 expression and inhibits migration. This mechanism involves downregulation of AKT, NF-κB, and JAK/STAT signaling pathways, along with suppressed MMP-2/MMP-9 activation [52]. Sesamol, a low-molecular-weight sesame-related phenol, exhibits a pronounced dose-dependent antitumor phenotype. At lower concentrations, it primarily functions as a free radical scavenger, whereas at higher exposure levels, it shifts to pro-oxidative activity and triggers mitochondrial apoptosis pathways [38]. In colorectal cancer HCT116 cells, sesamol significantly reduced cell survival and disrupted cell cycle progression within the 0.5–5 mM range, causing S-phase arrest. Concurrently, intracellular superoxide anion levels increased, accompanied by decreased mitochondrial membrane potential, DNA fragmentation, and nuclear morphological alterations, ultimately inducing apoptosis. In summary, existing studies suggest that the mechanisms of sesame lignans can be categorized into three recurring modules: First, p53/PTEN-AKT-related cellular regulation, corresponding to proliferation inhibition and apoptosis induction; second, the hypoxia–angiogenesis circuit mediated by IκBα/NF-κB-HIF-1α-VEGFA; third, p38/JNK-MMP-related migration and invasion regulation, which intersects with immune escape marker regulation (e.g., PD-L1) in certain models.

#### 2.1.4. Piper Lignans (Cubeba/Hinokinin)

Gusson-Zanetoni et al. systematically evaluated the effects of crude hydroalcoholic extracts from *Piper cubeba* fruit and their isolated lignans on cellular behaviors such as growth and migration while simultaneously observing changes in molecules associated with inflammation and tumor invasion [53]. The study employed Hep-2 laryngeal squamous cell carcinoma cells and SCC-25 oral squamous cell carcinoma cells, with normal fibroblasts serving as non-tumor controls. Evaluations were conducted across cellular morphology, proliferation, migration phenotypes, and cytotoxicity/genotoxicity. Results showed that the total extract and multiple lignans did not significantly alter overall cell morphology. However, they reduced tumor cell proliferation and migration under different time and dose conditions. The authors suggest these phenotypic effects may relate to regulated molecular expression in pathways associated with inflammation and invasion. On one hand, PTGS2 (COX-2) and PGE2 receptors (PTGER3/PTGER4), key nodes in inflammatory mediator synthesis and signaling, are frequently associated with tumor cell proliferation, survival, and migration phenotypes. On the other hand, MMP-2/MMP-9, belonging to the matrix metalloproteinase family, participate in extracellular matrix remodeling and influence cell adhesion and migration. Overall, this work provides comprehensive phenotypic evidence for the in vitro activity of *P. cubeba* lignans in a head and neck squamous cell carcinoma model. Another study using *P. cubeba* fruit as the source further advanced the mechanism to the cellular death process itself: After isolating and identifying 16 compounds, the authors found that chloroform fractions exhibited more pronounced inhibition against breast cancer cells (MCF-7) [40]. Among these, the lignan hinokinin induced approximately 40–50% proliferation inhibition at 10 μM. In MCF-7 cells, hinokinin was shown to trigger apoptosis via a mitochondrial dysfunction-centered pathway by inducing loss of mitochondrial membrane potential through elevated ROS levels. Based on these findings, Gusson-Zanetoni et al. emphasized the effects of *P. cubeba* lignans on tumor cell growth/migration phenotypes and inflammation-invasion-related factors, while hinokinin-related studies provided direct evidence of its antitumor effects through ROS-induced mitochondrial imbalance apoptosis in specific breast cancer models. Together, these studies demonstrate that *P. cubeba* lignans simultaneously modulate invasion-related phenotypes and mitochondrial stress across different tumor types, establishing a framework for future cross-validation studies exploring the COX-2/PGE2-MMP axis and mitochondrial pathways.

#### 2.1.5. Furofuran

Furofuran lignans are a class of structurally diverse lignan subclasses that have been reported to possess antioxidant, anti-inflammatory, cytotoxic, and antibacterial activities [54]. Pinoresinol, as a lignan phytoestrogen, exhibits consistent antitumor activity across breast cancer cells with different estrogen receptor statuses [41]. Antiproliferative effects were observed in both ER+ MCF-7 and ER− MDA-MB-231 cells. Conversely, it demonstrates an opposite response pattern in normal mammary epithelial cells (MCF10A), exhibiting greater antioxidant and cytoprotective effects. Notably, tumor cell survival rates decrease even at low doses (approximately 0.001–0.1 μM), while MCF10A cells show minimal changes. Under oxidative stress conditions, pinoresinol tends to promote oxidative regulation in tumor cells, increasing ROS levels and enhancing DNA damage signals. Conversely, in MCF10A cells, it alleviates oxidative stress-related DNA damage. This represents a clear example of a dual response, with tumor-suppressive effects in cancer cells and cytoprotective effects in non-tumor cells, potentially reflecting differences in redox vulnerability between tumor and normal cells. A similar dual effect is observed in another lignan, Schisandrin B. Within the doxorubicin chemotherapy system, it has been reported to selectively enhance doxorubicin-induced apoptosis in tumor cells without amplifying effects in normal cells (such as adult rat cardiomyocytes). This approach sensitizes tumors to chemotherapy while avoiding excessive damage to normal cells [42,43]. At the mechanistic level, this dual effect can often be explained by tumor cells’ higher baseline oxidative stress and more fragile compensatory capacity: the same compound tends to enhance antioxidant capacity in normal cells, thereby mitigating stress damage, whereas in tumor cells it is more likely to push ROS beyond the threshold or amplify mitochondrial-related damage, ultimately leading to sensitized apoptosis or growth inhibition [55].

#### 2.1.6. Neolignans

Honokiol and Magnolol are structurally classified as biphenyl neolignans derived from *Magnolia* (strictly speaking, they are not typical 8–8′ lignans), but since they share the same phenylpropane coupling origin as lignans, they are often discussed together in reviews of natural products for anticancer activity. Furthermore, they exhibit systematic in vitro and in vivo efficacy and mechanism evidence in colorectal cancer. Therefore, this paper includes them under the unified framework of “lignan/neolignan” and discusses them in the same section.

Honokiol has been demonstrated in multiple studies to inhibit the proliferation of colorectal cancer cells. In models characterized by CSC enrichment, honokiol significantly reduces spheroid formation capacity while downregulating markers associated with colorectal cancer stemness (such as DCLK1), suggesting its inhibitory effect on stemness maintenance [44]. This CSC phenotype is associated with sustained Notch signaling activation. Notch receptors require extracellular cleavage followed by critical cleavage by the γ-secretase complex to release active fragments into the nucleus. These fragments drive transcription programs involving Hes-1 and Cyclin D1, thereby maintaining stemness, promoting proliferation, and enhancing repair capacity. Previous studies indicate that honokiol can interfere with the proteolytic activation of Notch, inhibiting γ-secretase-related steps and reducing cleaved Notch and its downstream transcriptional output, thereby weakening Notch maintenance and tolerance in the context of CSC enrichment. Furthermore, in an AOM/DSS-induced colitis-associated carcinogenesis (CAC) model, oral administration of honokiol at 5 mg/kg three times weekly for 24 weeks following AOM injection and DSS cycles resulted in significant reductions in both the number and volume of colonic tumors. No notable changes in body weight or macroscopic abnormalities in major organs were observed [45]. Mechanistically, it suppresses the CSC phenotype by reducing colonosphere formation capacity and downregulating markers such as DCLK1/LGR5/CD44/SOX-9, suggesting potential direct inhibition of DCLK1 kinase activity while simultaneously restricting YAP1-TEAD transcriptional activity [45]. This promotes PUMA-YAP1 interaction, leading to cytoplasmic retention of YAP1 and downregulation of TEAD1/2/4. Consequently, in vivo studies demonstrate an overall reduction in YAP1/TEAD activity and CSC-related markers. Compared to honokiol, magnolol also possesses substantial evidence in colorectal cancer (CRC). Multiple studies indicate that magnolol inhibits the proliferation, migration, and invasion of colon cancer cells, demonstrating a clear antitumor effect in the HCT116 nude mouse xenograft model [46,47]. Mechanistic studies suggest its antitumor effects are associated with regulation of the Wnt/β-catenin pathway. Other research has also explained this from the perspective of cellular stress and death programs, such as through AMPK activation triggering apoptosis-related.

#### 2.1.7. Considerations for Anticancer Lignan Development

While many lignans have demonstrated promising anticancer activity in cellular experiments, further development requires more than just low IC_50_ values. Different lignan subclasses appear to function through distinct biological mechanisms. Aryltetrahydronaphthalene lignans, particularly podophyllotoxin derivatives, are relatively well-established examples, as their interactions with tubulin or topoisomerase II have been extensively studied. In contrast, other types of lignans, such as dibenzylbutyrolactone, arylnaphthalene, sesame, Piper-derived, and bisphenolic neolignan scaffolds, are generally associated with broader cellular effects, including apoptosis, inhibition of cell migration and invasion, regulation of oxidative stress, inhibition of angiogenesis, or modulation of cancer stem cell-related pathways. These findings are valuable, but they also indicate that the mechanism of action for many lignans still needs to be clarified in greater detail. For subsequent anticancer research, distinguishing between general cytotoxicity and selective activity is crucial. A compound that produces similar effects in normal cells, even if it reduces cancer cell viability, is not necessarily an effective lead compound. Therefore, studies should include non-tumor cell controls whenever possible, rather than simply comparing different cancer cell lines. In addition to viability assays, experiments related to apoptosis, cell cycle distribution, migration, invasion, intracellular reactive oxygen species levels, mitochondrial function, and related signaling proteins can help interpret whether observed activity is related to specific biological processes. Overall, the anticancer evidence for lignans encompasses a broad range of skeletal structures and biological endpoints, but the strength of evidence varies significantly across different branches. However, most studies remain confined to cellular experiments, with varying model comparability. Collectively, these studies confirm lignans as a rich source of anticancer lead structures while highlighting challenges requiring systematic resolution.

### 2.2. Antioxidant Activity

**Figure 4:**
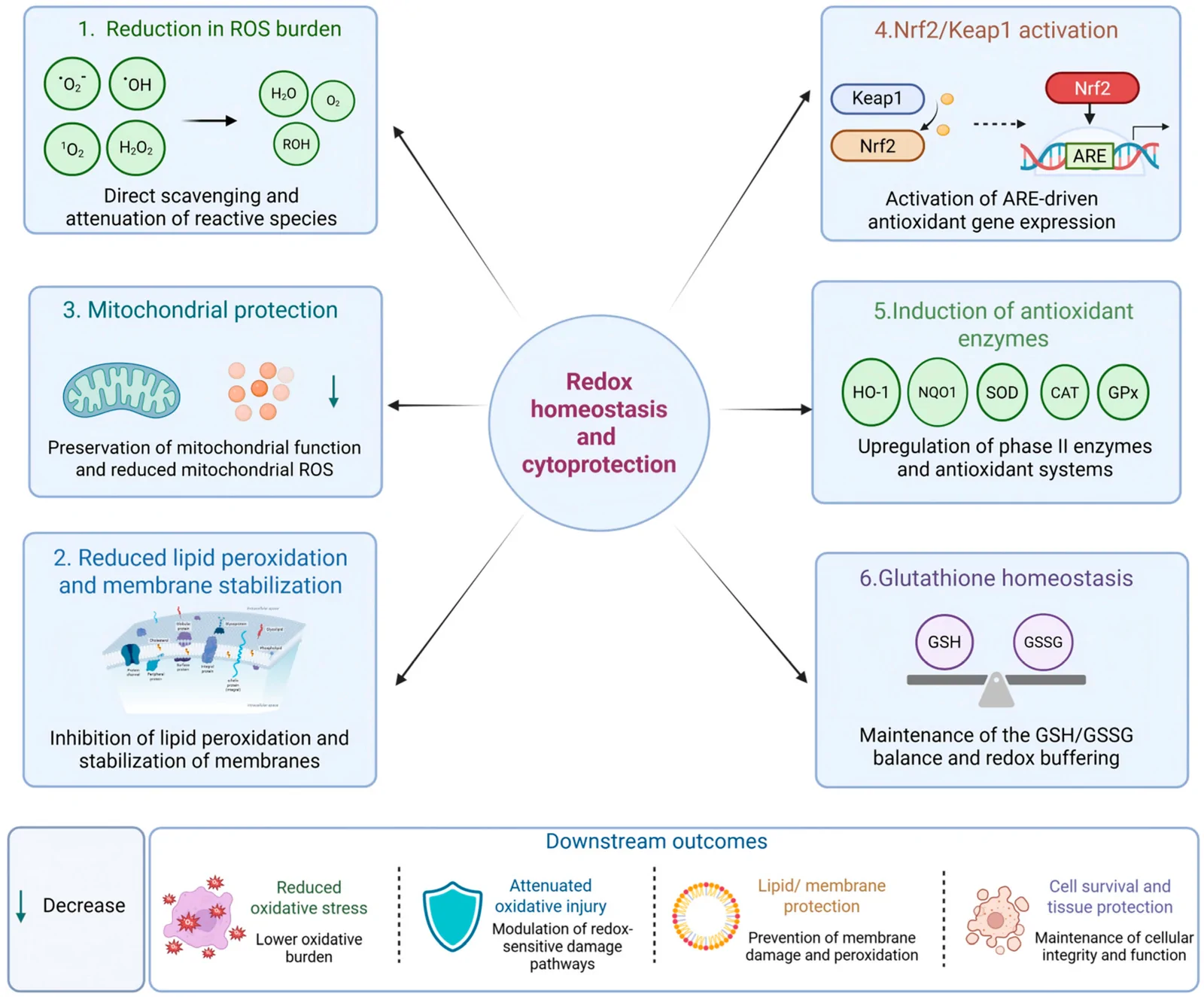
Antioxidant and redox-regulatory mechanisms of representative lignan subclasses. This schematic summarizes the main antioxidant mechanisms of representative lignan subclasses, including lipid protection, cytoprotective redox regulation, and Nrf2/Keap1-mediated antioxidant defense. Created in BioRender. https://BioRender.com/bcg3gy9.

Oxidative stress is a recurring biological theme associated with a wide range of disease states. It is not merely the result of tissue damage, inflammation, infection or metabolic imbalance but rather a dynamic factor that influences mitochondrial function, membrane stability, inflammatory signalling and cell survival. The excessive accumulation of reactive oxygen species (ROS) disrupts redox homeostasis; consequently, the pharmacological significance of antioxidant activity lies primarily in restoring or maintaining redox balance within an appropriate range. Many lignans contain phenolic, methoxy, or methylenedioxy substituents that can influence their redox behavior. The antioxidant activity of lignans is not limited to simple free radical scavenging; in many biological systems, it also involves the regulation of endogenous defence pathways, the maintenance of glutathione homeostasis, and the broader control of oxidative damage [56]. The antioxidant section of this review focuses not only on whether lignans exhibit antioxidant activity but also on how different structural branches contribute to redox processes in unique ways (Figure 4). In addition, some lignans may exhibit context-dependent or bidirectional redox behavior: under physiological or moderate stress conditions, they tend to enhance antioxidant defense and cell protection, while in tumor cells with high basal oxidative stress, this regulation may cause ROS to exceed the tolerable threshold and lead to apoptosis sensitization [57].

#### 2.2.1. Sesame Lignans

Sesame lignans, together with the related phenol sesamol, represent an important group of sesame-derived antioxidant compounds. Early mechanistic studies showed that sesaminol protects low-density lipoprotein from oxidative damage, demonstrating its antioxidant effect in a biologically relevant lipoprotein oxidation model [58]. Concurrently, sesamolin was reported to suppress lipid peroxidation in rat liver and kidney, extending the evidence to tissue-level oxidative injury in vivo [59]. Recent cellular studies have further demonstrated that sesamin and sesamol can alleviate oxidative stress induced by hydrogen peroxide in human neuronal cells via redox-related signalling pathways [60]. Taken together, these studies indicate that the antioxidant activity of sesame lignans is closely related to the control of lipid oxidative damage in physiologically meaningful systems, particularly those associated with membrane integrity and oxidative metabolic stress.

#### 2.2.2. Furofuran Lignans and Glycosides

Furofuran lignans and their glycosides further demonstrate that the antioxidant activity of the lignan family is closely related to disease-related cell cytoprotection. Compared to sesame lignans, they are more commonly observed in studies involving metabolic damage, neuroinflammation, and oxidative stress-related tissue dysfunction. For example, pinoresinol-4-O-β-D-glucopyranoside from *Prunus domestica* showed in vitro antioxidant activity and reduced oxidative stress markers in vivo [61,62]. This finding is significant because it places the antioxidant activity of this scaffold within a biologically relevant context, rather than simply limiting it to a chemical analysis. This study suggests that pinoresinol glycosides may exert their effects through a broader regulatory network associated with oxidative damage. Based on existing evidence, furofuran lignans and their glycosides are noteworthy because they link redox regulation to the maintenance of tissue function under metabolic or neurodegenerative stress.

#### 2.2.3. Dibenzocyclooctadiene Lignans

In parallel, dibenzocyclooctadiene lignans from *Schisandra*, particularly schisandrin B, are more strongly associated with endogenous antioxidant defense systems. Compared to the furofuran branch, the antioxidant evidence for dibenzocyclooctadiene lignans is more clearly linked to the regulation of pathway levels, particularly in the regulation of glutathione homeostasis and Nrf2-related signaling. Schisandrin B has been shown to elicit a glutathione antioxidant response and protect against apoptosis through the redox-sensitive ERK/Nrf2 pathway [63]. Subsequent studies further linked its protective effects to modulation of the Nrf2/Keap1-mediated antioxidant pathway under acute oxidative stress conditions [64]. This branch illustrates an important characteristic of lignan pharmacology: some compounds can not only neutralize ROS but also remodel stress sensitivity while protecting normal tissues in disease models. In this sense, dibenzocyclooctadiene lignans have pharmacological value because they build a clearer bridge between antioxidant phenotypes and well-defined intracellular signaling pathways.

Collectively, these antioxidant-related lignan branches differ in both their evidence patterns and mechanistic focus. Evidence for sesame lignans is most extensive in lipid-peroxidation and oxidative-damage models, supporting their well-established antioxidant properties. Furofuran lignans and their glycosides are more commonly associated with metabolic and neurodegenerative diseases, and have been observed to reduce oxidative stress, have anti-inflammatory effects, and provide tissue protection. In contrast, dibenzocyclooctadiene lignans show a clearer connection to endogenous antioxidant signaling. This comparison suggests that the antioxidant activity of lignans should be understood as a structurally differentiated pharmacological feature rather than a uniform biological activity.

### 2.3. Pharmacokinetic Considerations and Translational Relevance

Although lignans and neolignans exhibit a variety of different biological activities in cell and animal models, their translational potential is strongly influenced by pharmacokinetic behavior. Poor water solubility, limited intestinal absorption, extensive first-pass metabolism, and formulation dependence can all reduce the systemic concentration of the active parent compound. Therefore, the pharmacokinetic data summarized in Table 2 should be understood as representative examples related to exposure, rather than a directly comparable cross-skeletal ranking. Table 2 summarizes representative oral pharmacokinetic parameters reported for selected lignans and related compounds. Because the included studies differ in species, sex, dose, formulation, analytical method, and administered matrix, these values should be interpreted as representative exposure-related examples rather than as directly comparable cross-compound rankings.

## 3. Discussion

Lignans and related neolignans constitute a family with strong structural heterogeneity. Their pharmacological value does not stem from a single universal characteristic, such as a large number of oxygen-containing groups, complex stereochemistry, or the presence of multiple modifiable sites. Instead, the biological and pharmacokinetic behavior of individual compounds depends on the combined effects of factors such as skeleton type, substituent pattern, stereochemistry, metabolism, and formulation. In the field of anticancer drug development, a particularly important characteristic of certain lignans is their difference in effects on tumor cells and non-tumor cells. This distinction is crucial because if similar toxicity is observed in normal cells, then even strong cytotoxicity against cancer cells would have limited clinical value. Pinoresinol is a good example: in breast cancer models, it exhibited cytotoxicity, antiproliferative activity, and pro-oxidative effects on MCF-7 and MDA-MB-231 cells, while showing lower sensitivity in non-tumor cells such as MCF10A mammary epithelial cells, and exhibited antioxidant activity, resisting oxidative DNA damage [41]. Similarly, schisandrin B enhanced doxorubicin-induced apoptosis in cancer cells without increasing apoptosis in primary cardiomyocytes or human fibroblasts. Subsequent in vivo studies showed that schisandrin B, while maintaining or enhancing anticancer activity, also protected the heart from chronic doxorubicin toxicity [42,43]. These examples suggest that tumor-selective activity and protection of normal cells may be more therapeutically significant than any single structural feature.

Podophyllotoxin is primarily associated with inhibition of tubulin polymerization and disruption of mitotic microtubule dynamics, whereas the semisynthetic epipodophyllotoxin derivatives etoposide and teniposide predominantly act by stabilizing topoisomerase II–DNA cleavage complexes [16,17,19]. Importantly, early structure–activity studies showed that modification at the C-4 position can shift the balance between these two activities. Liu et al. reported that selected C-4 β-substituted podophyllotoxin analogues displayed stronger inhibition of DNA topoisomerase II but weaker inhibition of tubulin polymerization than corresponding compounds with different stereochemical configurations [70,71]. More recent studies indicate that C-4 modification does not necessarily produce a simple switch between tubulin and topoisomerase II activity. For example, the 4β-N-substituted podophyllotoxin derivative 5p displayed dual activity, acting as a catalytic inhibitor of topoisomerase IIαwhile also inhibiting microtubule polymerization, and retained activity against multidrug-resistant cancer models [72]. Therefore, in aryltetralin lignans, stereochemistry is pharmacologically relevant because specific stereochemical and substituent changes can alter target recognition and consequently shift the dominant mechanism of action.

A second important structure–activity relationship concerns the chemical form and position of oxygen-containing substituents. A free phenolic hydroxyl group can act as a hydrogen donor in radical-scavenging reactions and can increase the hydrogen-bonding capacity of a molecule, whereas O-methylation removes this hydrogen-donating function and may alter lipophilicity and metabolic susceptibility. However, the overall effect on aqueous solubility cannot be predicted solely from the number of hydroxyl or methoxy groups; molecular size, aromatic surface area, ionization, and conformation also contribute. In the *Justicia procumbens* series, hydroxyl substitution at C-1 and C-6′ was associated with increased antiproliferative and apoptogenic activity, whereas methoxylation at C-1 reduced activity [35]. These results suggest that the position and chemical form of oxygen substituents may be more important than a simple skeletal description. This distinction is also evident in sesame-derived compounds. Sesamol contains a free phenolic hydroxyl group and shows direct radical-scavenging behavior, whereas sesamin and sesamolin lack a free phenolic hydroxyl group and display a different antioxidant profile. In a comparative lipid-peroxidation study, sesamol directly inhibited oxidation in both microsomal and mitochondrial systems, while the antioxidant effects of sesamin and sesamolin were more dependent on the experimental system and were proposed to involve metabolic conversion [73]. This comparative result shows that direct antioxidant activity depends on specific functional groups, rather than simply the total oxygen content of the molecule.

Glycosylation represents another structural modification. Addition of a sugar moiety increases molecular polarity and hydrogen-bonding capacity, but it also increases molecular size and may reduce passive membrane permeability. Intact glycosides may not always represent the primary pharmacologically relevant forms in vivo. Many plant lignan glycosides are hydrolyzed in the gastrointestinal tract and subsequently metabolized by microorganisms [74,75]. Arctiin is a prime example. Human gut bacteria can convert arctiin to arctigenin, which further generates other demethylated and dehydroxylated metabolites [76]. Therefore, glycosylation not only affects its behavior in aqueous solution but also the types and timing of its entry into systemic circulation. The relationship between structure and pharmacokinetics is similarly compound-specific. Sesamin shows very low systemic exposure after oral administration as a conventional suspension, with an absolute bioavailability of approximately 0.3%, whereas formulation in a self-nanoemulsifying drug delivery system increased intestinal permeability and raised absolute bioavailability to approximately 4.4% [65]. The introduction of a phosphate group into etoposide can generate the water-soluble prodrug etoposide phosphate, which is rapidly converted to etoposide in vivo [77]. Honokiol and magnolol further illustrate that similar functional-group composition does not necessarily result in similar exposure; despite being closely related bisphenolic neolignans, magnolol showed substantially higher oral exposure than honokiol under matched emulsion-based conditions [69]. These examples demonstrate that the solubility, permeability, metabolism, and systemic exposure of individual lignans should be assessed, rather than inferred from general descriptors such as oxygen content or structural complexity.

## 4. Conclusions

Lignans and related neolignans constitute a structurally diverse family of natural products with broad anticancer and antioxidant potential. Among them, aryltetralin lignans remain the most informative mechanistic reference scaffold because they connect natural-product chemistry, clinically validated derivatives, and clearly defined molecular pharmacology. By contrast, many non-aryltetralin branches provide complementary phenotypes, including anti-metastatic, redox-regulatory, mitochondrial stress-modulating, and cytoprotective effects.

A major conclusion of this review is that the pharmacological value of lignans cannot be adequately understood through structural cataloguing or activity listing alone. More meaningful comparisons emerge when lignan branches are evaluated according to evidence strength, mechanistic anchoring, pharmacokinetic behavior, and development relevance. Under this framework, different scaffolds appear to map onto distinct opportunity spaces: aryltetralin lignans serve most strongly as anticancer reference scaffolds; arylnaphthalene lignan lactones, dibenzylbutyrolactone lignans, sesame lignans, Piper-derived lignans, and bisphenolic neolignans provide complementary anticancer mechanisms; and furofuran, sesame, and dibenzocyclooctadiene lignans are particularly relevant to redox regulation and antioxidant defense.

Future progress in this field will depend not only on reporting more activity data but also on building standardized evidence chains that connect scaffold class, mechanism, exposure-related properties, metabolite formation, and in vivo relevance. This will be important for moving lignan research from descriptive natural-product studies toward a more mechanism-based framework for medicinal chemistry and drug discovery.

## Figures and Tables

**Table 1:** Anticancer activity landscape of representative lignan and neolignan branches.

Structural Branch	Compound	Dose/ Concentration	Experimental Model	IC_50_	Mechanisms	Ref.
Aryltetralin lignans	Podophyllotoxin	Increasing concentrations; 6 μM tubulin-GTP	In vitro tubulin polymerization assay	IC_50_ = 4.19 μM	inhibition of microtubule polymerization	[16,26]
Aryltetralin lignans	Etoposide	Concentration-response assays	Topo IIα biochemical assay; DU145, HeLa, and A549 cancer cells	Topo IIα IC_50_ = 47.5 ± 2.2 μM; cellular IC_50_ = 1.0 ± 0.8 μM (DU145), 2.4 ± 0.9 μM (HeLa), and 1.3 ± 0.1 μM (A549)	Stabilization of Topo II–DNA cleavage complexes; DNA damage	[17,19,27]
Dibenzylbutyrolactone lignans	Arctigenin	50 mg/kg/day, p.o., 2 weeks	CT26 lung metastasis mouse model	Pulmonary tumor nodules reduced by approximately 30%; IC_50_ not reported in this study	Apoptosis; EMT suppression; ↓MMP-2/MMP-9	[28]
Dibenzylbutyrolactone lignans	Tracheloside	1–100 μM in vitro; 25 or 50 mg/kg/day in vivo	CT26, SW480, and SW620 CRC cells; CT26 lung-metastasis model	At 48 h, total apoptotic CT26 cells were approximately 10.0%, 15.5%, and 18.1% at 10, 50, and 100 μM, respectively; IC_50_ not reported	G0/G1 arrest; mitochondrial apoptosis; EMT/MMP regulation	[29]
Dibenzylbutyrolactone lignans	(−)-Trachelogenin	Concentration-response assay; 5–10 μM used for autophagy studies	HCT-116 and other tumor/non-tumor cell lines	IC_50_ = 1.9 μM in HCT-116; tumor-cell IC_50_ range 0.8–32.4 μM; IC_50_ > 64 μM in tested non-tumor cells	Autophagic cell death; ↑Beclin-1; ↑LC3-I to LC3-II conversion	[30,31]
Arylnaphthalene lignan lactones	Diphyllin	48 h concentration-response assay	TE-1 and ECA-109 esophageal cancer cells	IC_50_ = 0.2058 μM (TE-1) and 0.2792 μM (ECA-109)	V-ATPase inhibition; S-phase arrest; mTORC1/HIF-1α/VEGF-C suppression	[32,33]
Arylnaphthalene lignan lactones	6′-Hydroxyjusticidin B/Justicidin B	48 h concentration-response assay	K562 leukemia cells	IC_50_ = 20.0 μM for 6′-hydroxyjusticidin B and 45.4 μM for justicidin B	Caspase-dependent apoptosis; mitochondrial cell-death signaling	[34,35]
Sesame lignans	Sesamin	75 and 150 μM, 24 h	HeLa and SiHa cervical cancer cells	At 150 μM, apoptotic cells reached approximately 21.3% in HeLa and 36.5% in SiHa; IC_50_ not reported	p53/PUMA/Bax/PTEN activation; ↓AKT phosphorylation	[36,37]
Sesame-related phenol	Sesamol	0.5–5 mM for antiproliferative/pro-oxidant effects	HCT116 colorectal cancer cells	IC_50_ ≈ 2.59 mM; high concentrations induced S-phase arrest and apoptosis	ROS accumulation; mitochondrial dysfunction; DNA fragmentation	[38,39]
Piper lignans	Hinokinin	10 μM	MCF-7 and other human cancer cell lines	Approximately 40–50% growth inhibition was reported among the most responsive tested cancer-cell systems; IC_50_ not reported	ROS generation; loss of mitochondrial membrane potential; apoptosis	[40]
Furofuran lignans	(+)-Pinoresinol	0.001–100 μM, 24 h	MDA-MB-231, MCF-7, and non-tumorigenic MCF10A cells	At 0.001 μM, cell death was approximately 29% in MDA-MB-231, 20% in MCF-7, and 10% in MCF10A; IC_50_ not reported	Differential redox regulation; oxidative stress and DNA damage in tumor cells	[41]
Dibenzocyclooctadiene lignans	Schisandrin B	50 μM with doxorubicin	SMMC7721 and MCF-7 cancer cells; primary cardiomyocytes/fibroblasts	50 μM was selected as a minimally cytotoxic concentration	Chemosensitization; mitochondrial apoptosis; redox modulation	[42,43]
Bisphenolic neolignans	Honokiol	25 μM in vitro; 5 mg/kg, p.o., 3 times/week for 24 weeks in vivo	HCT116/SW480 CRC cells; AOM/DSS colitis-associated cancer model	5 mg/kg significantly reduced colonic tumor number and size	DCLK1/CSC suppression; YAP1/TEAD regulation; apoptosis	[44,45]
Bisphenolic neolignans	Magnolol	12.5–75 μM in vitro; 5 mg/kg, i.p., 3 times/week for 20 days	HCT116/SW480 CRC cells; HCT116 xenograft model	54.6% inhibition of xenograft tumor growth at 5 mg/kg; IC_50_ not reported in the selected CRC study	Wnt/β-catenin signaling; inhibition of proliferation, invasion, and motility	[46,47]

**Table 2:** Representative pharmacokinetic parameters of selected lignans and related compounds.

Compound	Structural Branch	Species/ Model	Oral Administration and Formulation	Cmax (μg/mL)	Tmax (h)	t1/2 (h)	AUC (μg·h/mL)	F (%)	Key Pharmacokinetic Observation	Ref.
**Sesamin**	Sesame lignan	Male Sprague-Dawley rat	100 mg/kg, p.o., suspension in 2% carboxymethyl cellulose	0.03 ± 0.004	NR	4.7 ± 3.1	0.13 ± 0.03	0.3	Unchanged sesamin showed very low systemic exposure.	[65]
**Sesamin**	Sesame lignan	Male Sprague-Dawley rat	100 mg/kg, p.o., self-nanoemulsifying drug delivery system (SNEDDS)	0.23 ± 0.02	NR	10.5 ± 2.4	1.70 ± 0.63	4.4	SNEDDS increased Cmax, AUC0–t, and absolute oral bioavailability relative to the suspension.	[66]
**Sesamol**	Sesame-related phenol	Rat	50 mg/kg, Gavage	0.44 ± 0.001	2.00 ± 0.04	10.9 ± 0.06	6.01 ± 0.004	35.5 ± 8.5	Rapid absorption and broad tissue distribution were reported in this solution-based study.	[66]
**Arctigenin**	Dibenzylbutyrolactone lignan	Piglet	1.0 g/kg, p.o., *Fructus arctii* powder	0.43 ± 0.04	0.85 ± 0.21	63.47 ± 29.12	14.67 ± 4.81	NR	A bimodal plasma profile was observed after plant-powder administration; exposure may reflect both arctigenin and arctiin conversion.	[67]
**Schisandrin B**	Dibenzocyclooctadiene lignan	Female and male Sprague-Dawley rat	p.o., 10–40 mg/kg, micronized particles	NR	NR	NR	NR	55.0 (female); 19.3 (male)	Absolute oral bioavailability was sex-dependent; dose-linear pharmacokinetics were reported over 10–40 mg/kg.	[68]
**Magnolol**	Bisphenolic neolignan	Male Sprague-Dawley rat	50 mg/kg, p.o., isomer emulsion	0.43 ± 0.27	1.20	NR	NR	17.5 ± 9.7	Magnolol showed higher oral exposure than honokiol under matched emulsion-based conditions.	[69]
**Honokiol**	Bisphenolic neolignan	Male Sprague-Dawley rat	50 mg/kg, p.o., isomer emulsion	0.04 ± 0.03	0.45	NR	NR	5.3 ± 11.7	Honokiol showed lower Cmax and lower absolute oral bioavailability than magnolol under matched conditions.	[69]

Abbreviations: Cmax, maximum plasma concentration; Tmax, time to maximum plasma concentration; t1/2, elimination half-life; AUC, area under the plasma concentration–time curve; F, absolute oral bioavailability; NR, not reported.

## Data Availability

No new data were created or analyzed in this study. Data sharing is not applicable to this article.

## Abbreviations

The following abbreviations are used in this manuscript:

AKT	Protein kinase B
AMPK	AMP-activated protein kinase
AOM	Azoxymethane
AOM/DSS	Azoxymethane/dextran sulfate sodium
AVOs	Acidic vesicular organelles
CAC	Colitis-associated carcinogenesis
CCK-8	Cell Counting Kit-8
CD44	Cluster of differentiation 44
CDK4	Cyclin-dependent kinase 4
CME	Chinensinaphthol methyl ether
COX-2	Cyclooxygenase-2
CRC	Colorectal cancer
CSC	Cancer stem cell
DCLK1	Doublecortin-like kinase 1
DBBL	Dibenzylbutyrolactone
DPT	Deoxypodophyllotoxin
DPT-HP-β-CD	Deoxypodophyllotoxin/2-hydroxypropyl-β-cyclodextrin complex
DSS	Dextran sulfate sodium
EMT	Epithelial–mesenchymal transition
ER	Estrogen receptor
HIF-1α	Hypoxia-inducible factor 1-alpha
HP-β-CD	2-Hydroxypropyl-β-cyclodextrin
IκBα	Inhibitor of κB alpha
JAK/STAT	Janus kinase/signal transducer and activator of transcription
JNK	c-Jun N-terminal kinase
Keap1	Kelch-like ECH-associated protein 1
LC3-I/LC3-II	Microtubule-associated protein 1 light chain 3 forms I and II
LGR5	Leucine-rich repeat-containing G-protein-coupled receptor 5
MAPK	Mitogen-activated protein kinase
MMP	Matrix metalloproteinase
MMP-2/MMP-9	Matrix metalloproteinase-2/-9
mTORC1	Mechanistic target of rapamycin complex 1
MTT	3-(4,5-Dimethylthiazol-2-yl)-2,5-diphenyltetrazolium bromide
NF-κB	Nuclear factor kappa B
Nrf2	Nuclear factor erythroid 2-related factor 2
PD-L1	Programmed death-ligand 1
PGE_2_	Prostaglandin E_2_
PTEN	Phosphatase and tensin homolog
PTGER3/PTGER4	Prostaglandin E receptor 3/4
PTGS2	Prostaglandin-endoperoxide synthase 2
PUMA	p53 upregulated modulator of apoptosis
ROS	Reactive oxygen species
SAR	Structure–activity relationship
SIRT1/SIRT3	Sirtuin 1/3
SOX-9	SRY-box transcription factor 9
TCS	Tracheloside
TEAD	TEA domain transcription factor
TEME	Taiwanin E methyl ether
Topo II	Topoisomerase II
UDP	Uridine diphosphate
V-ATPase	Vacuolar-type H^+^-ATPase
VEGFA	Vascular endothelial growth factor A
VEGF-C	Vascular endothelial growth factor C
VP-16	Etoposide
YAP1	Yes-associated protein 1

## References

[1] Barker D. (2019). Lignans. Molecules.

[2] Teponno R.B., Kusari S., Spiteller M. (2016). Recent advances in research on lignans and neolignans. Nat. Prod. Rep..

[3] Zálešák F., Bon D.J.D., Pospíšil J. (2019). Lignans and Neolignans: Plant secondary metabolites as a reservoir of biologically active substances. Pharmacol. Res..

[4] Wang L.X., Wang H.L., Huang J., Chu T.Z., Peng C., Zhang H., Chen H.L., Xiong Y.A., Tan Y.Z. (2022). Review of lignans from 2019 to 2021: Newly reported compounds, diverse activities, structure-activity relationships and clinical applications. Phytochemistry.

[5] Yeung A.W.K., Tzvetkov N.T., Balacheva A.A., Georgieva M.G., Gan R.Y., Jozwik A., Pyzel B., Horbańczuk J.O., Novellino E., Durazzo A. (2020). Lignans: Quantitative Analysis of the Research Literature. Front. Pharmacol..

[6] Umezawa T. (2003). Diversity in lignan biosynthesis. Phytochem. Rev..

[7] Davin L.B., Wang H.B., Crowell A.L., Bedgar D.L., Martin D.M., Sarkanen S., Lewis N.G. (1997). Stereoselective bimolecular phenoxy radical coupling by an auxiliary (dirigent) protein without an active center. Science.

[8] Markulin L., Corbin C., Renouard S., Drouet S., Gutierrez L., Mateljak I., Auguin D., Hano C., Fuss E., Lainé E. (2019). Pinoresinol-lariciresinol reductases, key to the lignan synthesis in plants. Planta.

[9] Murata J., Ono E., Yoroizuka S., Toyonaga H., Shiraishi A., Mori S., Tera M., Azuma T., Nagano A.J., Nakayasu M. (2017). Oxidative rearrangement of (+)-sesamin by CYP92B14 co-generates twin dietary lignans in sesame. Nat. Commun..

[10] Ono E., Waki T., Oikawa D., Murata J., Shiraishi A., Toyonaga H., Kato M., Ogata N., Takahashi S., Yamaguchi M.A. (2020). Glycoside-specific glycosyltransferases catalyze regio-selective sequential glucosylations for a sesame lignan, sesaminol triglucoside. Plant J..

[11] Frezza C., Venditti A., Toniolo C., De Vita D., Franceschin M., Ventrone A., Tomassini L., Foddai S., Guiso M., Nicoletti M. (2020). Nor-Lignans: Occurrence in Plants and Biological Activities—A Review. Molecules.

[12] Gordaliza M., García P.A., del Corral J.M., Castro M.A., Gómez-Zurita M.A. (2004). Podophyllotoxin: Distribution, sources, applications and new cytotoxic derivatives. Toxicon.

[13] Imbert T.F. (1998). Discovery of podophyllotoxins. Biochimie.

[14] You Y. (2005). Podophyllotoxin derivatives: Current synthetic approaches for new anticancer agents. Curr. Pharm. Des..

[15] Lau W., Sattely E.S. (2015). Six enzymes from mayapple that complete the biosynthetic pathway to the etoposide aglycone. Science.

[16] Jordan M.A., Wilson L. (2004). Microtubules as a target for anticancer drugs. Nat. Rev. Cancer.

[17] Hande K.R. (1998). Etoposide: Four decades of development of a topoisomerase II inhibitor. Eur. J. Cancer.

[18] Bray F., Laversanne M., Sung H., Ferlay J., Siegel R.L., Soerjomataram I., Jemal A. (2024). Global cancer statistics 2022: GLOBOCAN estimates of incidence and mortality worldwide for 36 cancers in 185 countries. CA Cancer J. Clin..

[19] Nitiss J.L. (2009). Targeting DNA topoisomerase II in cancer chemotherapy. Nat. Rev. Cancer.

[20] Pommier Y., Leo E., Zhang H., Marchand C. (2010). DNA topoisomerases and their poisoning by anticancer and antibacterial drugs. Chem. Biol..

[21] Khaled M., Belaaloui G., Jiang Z.Z., Zhu X., Zhang L.Y. (2016). Antitumor effect of Deoxypodophyllotoxin on human breast cancer xenograft transplanted in BALB/c nude mice model. J. Infect. Chemother..

[22] Botta B., Delle Monache G., Misiti D., Vitali A., Zappia G. (2001). Aryltetralin lignans: Chemistry, pharmacology and biotransformations. Curr. Med. Chem..

[23] Zhang X., Rakesh K.P., Shantharam C.S., Manukumar H.M., Asiri A.M., Marwani H.M., Qin H.L. (2018). Podophyllotoxin derivatives as an excellent anticancer aspirant for future chemotherapy: A key current imminent needs. Bioorg. Med. Chem..

[24] Motyka S., Jafernik K., Ekiert H., Sharifi-Rad J., Calina D., Al-Omari B., Szopa A., Cho W.C. (2023). Podophyllotoxin and its derivatives: Potential anticancer agents of natural origin in cancer chemotherapy. Biomed. Pharmacother..

[25] Tailor D., Going C.C., Resendez A., Kumar V., Nambiar D.K., Li Y., Dheeraj A., LaGory E.L., Ghoochani A., Birk A.M. (2021). Novel Aza-podophyllotoxin derivative induces oxidative phosphorylation and cell death via AMPK activation in triple-negative breast cancer. Br. J. Cancer.

[26] Sharma S., Ganesh T., Kingston D.G., Bane S. (2007). Promotion of tubulin assembly by poorly soluble taxol analogs. Anal. Biochem..

[27] Arencibia J.M., Brindani N., Franco-Ulloa S., Nigro M., Kuriappan J.A., Ottonello G., Bertozzi S.M., Summa M., Girotto S., Bertorelli R. (2020). Design, Synthesis, Dynamic Docking, Biochemical Characterization, and in Vivo Pharmacokinetics Studies of Novel Topoisomerase. J. Med. Chem..

[28] Han Y.H., Kee J.Y., Kim D.S., Mun J.G., Jeong M.Y., Park S.H., Choi B.M., Park S.J., Kim H.J., Um J.Y. (2016). Arctigenin Inhibits Lung Metastasis of Colorectal Cancer by Regulating Cell Viability and Metastatic Phenotypes. Molecules.

[29] Shin M.K., Jeon Y.D., Hong S.H., Kang S.H., Kee J.Y., Jin J.S. (2021). In Vivo and In Vitro Effects of Tracheloside on Colorectal Cancer Cell Proliferation and Metastasis. Antioxidants.

[30] Benvenuto M., Albonici L., Focaccetti C., Ciuffa S., Fazi S., Cifaldi L., Miele M.T., De Maio F., Tresoldi I., Manzari V. (2020). Polyphenol-Mediated Autophagy in Cancer: Evidence of In Vitro and In Vivo Studies. Int. J. Mol. Sci..

[31] Moura A.F., Lima K.S.B., Sousa T.S., Marinho-Filho J.D.B., Pessoa C., Silveira E.R., Pessoa O.D.L., Costa-Lotufo L.V., Moraes M.O., Araújo A.J. (2018). In vitro antitumor effect of a lignan isolated from Combretum fruticosum, trachelogenin, in HCT-116 human colon cancer cells. Toxicol. Vitr..

[32] Chen H., Liu P., Zhang T., Gao Y., Zhang Y., Shen X., Li X., Shen W. (2018). Effects of diphyllin as a novel V-ATPase inhibitor on TE-1 and ECA-109 cells. Oncol. Rep..

[33] Hou W., Huang L.-J., Huang H., Liu S.-L., Dai W., Li Z.-M., Zhang Z.-Y., Xin S.-Y., Wang J.-Y., Zhang Z.-Y. (2023). Bioactivities and Mechanisms of Action of Diphyllin and Its Derivatives: A Comprehensive Systematic Review. Molecules.

[34] Luo J., Qin J., Fu Y., Zhang S., Zhang X., Yang M. (2018). 6′-Hydroxy Justicidin B Triggers a Critical Imbalance in Ca(2+) Homeostasis and Mitochondrion-Dependent Cell Death in Human Leukemia K562 Cells. Front. Pharmacol..

[35] Luo J., Hu Y., Kong W., Yang M. (2014). Evaluation and Structure-Activity Relationship Analysis of a New Series of Arylnaphthalene lignans as Potential Anti-Tumor Agents. PLoS ONE.

[36] Kuo T.N., Lin C.S., Li G.D., Kuo C.Y., Kao S.H. (2020). Sesamin inhibits cervical cancer cell proliferation by promoting p53/PTEN-mediated apoptosis. Int. J. Med. Sci..

[37] Huang Y., Liu Z., Li L., Jiang M., Tang Y., Zhou L., Li J., Chen Y. (2022). Sesamin inhibits hypoxia-stimulated angiogenesis via the NF-κB p65/HIF-1α/VEGFA signaling pathway in human colorectal cancer. Food Funct..

[38] Wu M.S., Aquino L.B.B., Barbaza M.Y.U., Hsieh C.L., Castro-Cruz K.A., Yang L.L., Tsai P.W. (2019). Anti-Inflammatory and Anticancer Properties of Bioactive Compounds from Sesamum indicum L.-A Review. Molecules.

[39] Khamphio M., Barusrux S., Weerapreeyakul N. (2016). Sesamol induces mitochondrial apoptosis pathway in HCT116 human colon cancer cells via pro-oxidant effect. Life Sci..

[40] Lone B.A., Hossain M.M., Rani D., Shahi A., Sharma N., Gairola S., Gupta P. (2025). Hinokinin, a lignan from Piper cubeba fruits induces apoptosis via reactive oxygen species-mediated mitochondrial dysfunction in MCF-7 breast cancer cells. Nat. Prod. Res..

[41] López-Biedma A., Sánchez-Quesada C., Beltrán G., Delgado-Rodríguez M., Gaforio J.J. (2016). Phytoestrogen (+)-pinoresinol exerts antitumor activity in breast cancer cells with different oestrogen receptor statuses. BMC Complement. Altern. Med..

[42] Li L., Lu Q., Shen Y., Hu X. (2006). Schisandrin B enhances doxorubicin-induced apoptosis of cancer cells but not normal cells. Biochem. Pharmacol..

[43] Xu Y., Liu Z., Sun J., Pan Q., Sun F., Yan Z., Hu X. (2011). Schisandrin B prevents doxorubicin-induced chronic cardiotoxicity and enhances its anticancer activity in vivo. PLoS ONE.

[44] Ponnurangam S., Mammen J.M., Ramalingam S., He Z., Zhang Y., Umar S., Subramaniam D., Anant S. (2012). Honokiol in combination with radiation targets notch signaling to inhibit colon cancer stem cells. Mol. Cancer Ther..

[45] Subramaniam D., Ponnurangam S., Ramalingam S., Kwatra D., Dandawate P., Weir S.J., Umar S., Jensen R.A., Anant S. (2021). Honokiol Affects Stem Cell Viability by Suppressing Oncogenic YAP1 Function to Inhibit Colon Tumorigenesis. Cells.

[46] Park J.B., Lee M.S., Cha E.Y., Lee J.S., Sul J.Y., Song I.S., Kim J.Y. (2012). Magnolol-induced apoptosis in HCT-116 colon cancer cells is associated with the AMP-activated protein kinase signaling pathway. Biol. Pharm. Bull..

[47] Kang Y.J., Park H.J., Chung H.J., Min H.Y., Park E.J., Lee M.A., Shin Y., Lee S.K. (2012). Wnt/β-catenin signaling mediates the antitumor activity of magnolol in colorectal cancer cells. Mol. Pharmacol..

[48] Solyomváry A., Beni S., Boldizsar I. (2017). Dibenzylbutyrolactone Lignans—A Review of Their Structural Diversity, Biosynthesis, Occurrence, Identification and Importance. Mini Rev. Med. Chem..

[49] Xiao L., Chen X.-J., Feng J.-K., Li W.-N., Yuan S., Hu Y. (2023). Natural products as the calcium channel blockers for the treatment of arrhythmia: Advance and prospect. Fitoterapia.

[50] He M., Hu J., Deng J., Chen X., Joshua Olatunji O., Jayeoye T.J. (2025). A comprehensive review on the anti-cancer properties of sesame lignans: Chemical composition, molecular mechanisms and prospects. RSC Adv..

[51] Majdalawieh A.F., Massri M., Nasrallah G.K. (2017). A comprehensive review on the anti-cancer properties and mechanisms of action of sesamin, a lignan in sesame seeds (Sesamum indicum). Eur. J. Pharmacol..

[52] Chen J.M., Chen P.Y., Lin C.C., Hsieh M.C., Lin J.T. (2020). Antimetastatic Effects of Sesamin on Human Head and Neck Squamous Cell Carcinoma through Regulation of Matrix Metalloproteinase-2. Molecules.

[53] Gusson-Zanetoni J.P., da Silva J., Picão T.B., Cardin L.T., Prates J., Sousa S.O., Henrique T., Oliani S.M., Tajara E.H., E Silva M.L.A. (2022). Effect of Piper cubeba total extract and isolated lignans on head and neck cancer cell lines and normal fibroblasts. J. Pharmacol. Sci..

[54] Xu W.H., Zhao P., Wang M., Liang Q. (2019). Naturally occurring furofuran lignans: Structural diversity and biological activities. Nat. Prod. Res..

[55] Ong C.P., Lee W.L., Tang Y.Q., Yap W.H. (2019). Honokiol: A Review of Its Anticancer Potential and Mechanisms. Cancers.

[56] Tu W., Wang H., Li S., Liu Q., Sha H. (2019). The Anti-Inflammatory and Anti-Oxidant Mechanisms of the Keap1/Nrf2/ARE Signaling Pathway in Chronic Diseases. Aging Dis..

[57] Hassanein E.H.M., Althagafy H.S., Baraka M.A., Abd-alhameed E.K., Ibrahim I.M., Abd El-Maksoud M.S., Mohamed N.M., Ross S.A. (2024). The promising antioxidant effects of lignans: Nrf2 activation comes into view. Naunyn-Schmiedeberg’s Arch. Pharmacol..

[58] Kang M.H., Naito M., Sakai K., Uchida K., Osawa T. (2000). Mode of action of sesame lignans in protecting low-density lipoprotein against oxidative damage in vitro. Life Sci..

[59] Kang M.H., Naito M., Tsujihara N., Osawa T. (1998). Sesamolin inhibits lipid peroxidation in rat liver and kidney. J. Nutr..

[60] Ruankham W., Suwanjang W., Wongchitrat P., Prachayasittikul V., Prachayasittikul S., Phopin K. (2021). Sesamin and sesamol attenuate H(2)O(2)-induced oxidative stress on human neuronal cells via the SIRT1-SIRT3-FOXO3a signaling pathway. Nutr. Neurosci..

[61] Youssef F.S., Ashour M.L., El-Beshbishy H.A., Ahmed Hamza A., Singab A.N.B., Wink M. (2020). Pinoresinol-4-O-β-D-glucopyranoside: A lignan from prunes (Prunus domestica) attenuates oxidative stress, hyperglycaemia and hepatic toxicity in vitro and in vivo. J. Pharm. Pharmacol..

[62] Lei S., Wu S., Wang G., Li B., Liu B., Lei X. (2021). Pinoresinol diglucoside attenuates neuroinflammation, apoptosis and oxidative stress in a mice model with Alzheimer’s disease. Neuroreport.

[63] Leong P.K., Chiu P.Y., Chen N., Leung H., Ko K.M. (2011). Schisandrin B elicits a glutathione antioxidant response and protects against apoptosis via the redox-sensitive ERK/Nrf2 pathway in AML12 hepatocytes. Free Radic. Res..

[64] Wu Y., Li Z.C., Yao L.Q., Li M., Tang M. (2019). Schisandrin B alleviates acute oxidative stress via modulation of the Nrf2/Keap1-mediated antioxidant pathway. Appl. Physiol. Nutr. Metab..

[65] Wang C.-Y., Yen C.-C., Hsu M.-C., Wu Y.-T. (2020). Self-Nanoemulsifying Drug Delivery Systems for Enhancing Solubility, Permeability, and Bioavailability of Sesamin. Molecules.

[66] Geetha T., Singh N., Deol P.K., Kaur I.P. (2015). Biopharmaceutical profiling of sesamol: Physiochemical characterization, gastrointestinal permeability and pharmacokinetic evaluation. RSC Adv..

[67] He B., Zhang H.J., Yang W.H., Shao Z.Y., Wu L.J., Chen X.B., Chen J., Liu W., Ran Z.P., Jin R.G. (2019). Pharmacokinetics of Arctigenin and Fructus Arctii Powder in Piglets. Front. Vet. Sci..

[68] Wang Z., You L., Cheng Y., Hu K., Wang Z., Cheng Y., Yang J., Yang Y., Wang G. (2018). Investigation of pharmacokinetics, tissue distribution and excretion of schisandrin B in rats by HPLC-MS/MS. Biomed. Chromatogr..

[69] Sheng Y.L., Xu J.H., Shi C.H., Li W., Xu H.Y., Li N., Zhao Y.Q., Zhang X.R. (2014). UPLC-MS/MS-ESI assay for simultaneous determination of magnolol and honokiol in rat plasma: Application to pharmacokinetic study after administration emulsion of the isomer. J. Ethnopharmacol..

[70] Liu S.Y., Hwang B.D., Haruna M., Imakura Y., Lee K.H., Cheng Y.C. (1989). Podophyllotoxin analogs: Effects on DNA topoisomerase II, tubulin polymerization, human tumor KB cells, and their VP-16-resistant variants. Mol. Pharmacol..

[71] Lu B., Xu L., Chen H., Yu G., Khow K., Yang S., Dinker A., Njoo E. (2026). C-4 analogues of podophyllotoxin as tubulin inhibitors: Synthesis, biological evaluation, and structure-activity relationship. Nat. Prod. Res..

[72] Lv X., Cheng W.H., Li X.X., Shang H., Zhang J.Y., Hong H.Y., Zheng Y.J., Dong Y.Q., Gong J.H., Zheng Y.B. (2024). Dual inhibition of topoisomerase II and microtubule of podophyllotoxin derivative 5p overcomes cancer multidrug resistance. Eur. J. Pharmacol..

[73] Ghafoorunissa, Hemalatha S., Rao M.V. (2004). Sesame lignans enhance antioxidant activity of vitamin E in lipid peroxidation systems. Mol. Cell. Biochem..

[74] Baldi S., Tristán Asensi M., Pallecchi M., Sofi F., Bartolucci G., Amedei A. (2023). Interplay between Lignans and Gut Microbiota: Nutritional, Functional and Methodological Aspects. Molecules.

[75] Landete J.M. (2012). Plant and mammalian lignans: A review of source, intake, metabolism, intestinal bacteria and health. Food Res. Int..

[76] Xie L.H., Ahn E.M., Akao T., Abdel-Hafez A.A., Nakamura N., Hattori M. (2003). Transformation of arctiin to estrogenic and antiestrogenic substances by human intestinal bacteria. Chem. Pharm. Bull..

[77] Brooks D.J., Srinivas N.R., Alberts D.S., Thomas T., Igwemzie L.M., McKinney L.M., Randolph J., Schacter L., Kaul S., Barbhaiya R.H. (1995). Phase I and pharmacokinetic study of etoposide phosphate. Anticancer Drugs.

